# Satellite telemetry reveals habitat selection decisions by black oystercatchers across seasonal, diel, and tidal cycles

**DOI:** 10.1002/ece3.9957

**Published:** 2023-04-07

**Authors:** Lena Ware, John Mark Hipfner, David J. Green

**Affiliations:** ^1^ Department of Biological Sciences, Centre for Wildlife Ecology Simon Fraser University Burnaby British Columbia Canada; ^2^ Environment and Climate Change Canada Canadian Wildlife Service, Northern Region Whitehorse Yukon Canada; ^3^ Environment and Climate Change Canada Science and Technology Branch, Pacific Wildlife Research Centre Delta British Columbia Canada

**Keywords:** Argos, indicator species, intertidal health, marine shoreline, recurse, shorebird

## Abstract

Habitat use of indicator species is used to prioritize management activities. However, habitat use can vary temporally in response to changes in predation risk and foraging rewards. We deployed satellite tags on 20 black oystercatchers (*Haematopus bachmani*) in four regions of British Columbia, Canada, to examine habitat use and selection decisions across seasonal, diel and tidal cycles. We characterized the shoreline in each region and used GLMMs to investigate how habitat characteristics influenced shoreline use by tracked birds. For individuals, we estimated home range size and the frequency key features of the shoreline were re‐visited. Black oystercatchers generally made greater‐than‐expected use of rocky islets and shoreline with freshwater outflows, less tree cover and greater intertidal area. However, while black oystercatchers preferred islets and shoreline with less tree cover at most/all time periods, they only exhibited preferences for greater intertidal area during low tides, and preferences for shoreline with freshwater outflows during the nonbreeding season, day, and high tides. Individual home ranges, on average, contained 46 km of shoreline (range: 12–156 km) and individuals used 10.4 km (range: 6.7–13.9 km). Individuals made greater use of larger islets with less tree cover that were closer to outflows, and greater use of outflows associated with larger streams, greater intertidal areas and gravel substrates. Black oystercatchers' habitat preferences likely reduce predation risk (rocky islets and shoreline with less tree cover) and increase foraging rewards (shoreline with freshwater outflows, greater intertidal area, and gravel substrates). However, habitat preferences appear sensitive to constraints on movement in the breeding season and changes in foraging rewards across the diel and tidal cycle, highlighting the importance of examining habitat use at multiple temporal scales. Black oystercatchers are considered indicators of rocky intertidal health; therefore, critical habitat is expected to be important for a suite of wildlife dependent on safe and productive coastline.

## Graphical abstract

We examined how habitat use of black oystercatchers varied in response to changes in predation risk and foraging rewards by tracking birds across seasonal, diel, and tidal cycles. Black oystercatchers used safe rocky islets and shoreline with less tree cover at most times and more dangerous shoreline with greater intertidal area and freshwater outflows during low and high tides, respectively, when foraging rewards at these sites are likely higher. Identifying critical habitat that is expected to be indicative of important habitat for coastal wildlife requires studying habitat use at different times.

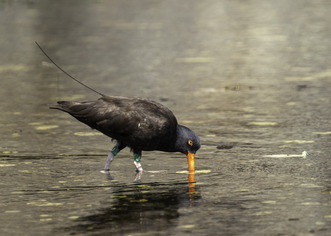

## 1 INTRODUCTION

Habitat selection decisions influence diet, energetic intake rates, survival, and reproductive success of individuals (Block & Brennan, 1993; Hutto, 1985). Resources, however, are unevenly distributed both in space and time requiring individuals to regularly update these decisions (Fretwell & Lucas, 1969; Railsback & Harvey, 2002). Individual habitat selection decisions can also be influenced by behavioral constraints, environmental stimuli, and trade‐offs and consequently reflect the changing risks and rewards to an individual (Block & Brennan, 1993; Mayor et al., 2009). For example, during a breeding season, movements are likely constrained by the need to provide parental care, so individuals may prioritize reproduction and select habitat that reduces nest depredation and increases offspring survival (Fontaine & Martin, 2006). During the nonbreeding season, individuals can move more widely and instead may prioritize the selection of habitat that enhances their own energetic intake, survival, and future reproductive success (Marra et al., 1998). Habitat selection decisions can also occur over finer temporal scales, such as day to night, in response to changes in predation risk and foraging conditions (Lima & Dill, 1990). Despite the spatiotemporal patterns of habitat use observed in many taxa, studies examining habitat selection decisions are frequently limited to one time period or season (Lunardi et al., 2012; Marra et al., 2015; Schooley, 1994; Specht et al., 2020). However, advances in tracking technology that allow smaller animals to be tracked for longer periods provide an opportunity to investigate individual movement and population‐level habitat selection decisions across the annual cycle (Cagnacci et al., 2010; Knight et al., 2021; Stanley et al., 2021).

The black oystercatcher (*Haematopus bachmani*) is a large shorebird restricted to marine shoreline habitat on the Pacific coast of North America, where they forage exclusively in the intertidal zone for macro‐invertebrate prey (Tessler et al., 2014). Suitable territories for nesting are limited because they must include physical features that provide protection from nest predators and nearby profitable foraging opportunities (Dalgarno et al., 2017). Breeding pairs consequently aggressively defend their territories during the spring and summer months (McFarland, 2010), likely constraining their movement and habitat selection decisions. Previous research on habitat use and preferences of black oystercatchers has been conducted almost exclusively during the breeding season. Studies have consistently found that nest‐site selection is influenced by habitat characteristics that reduce predation risk such as isolation from the mainland or distance from vegetation (Dalgarno et al., 2017; McFarland, 2010; Weinstein et al., 2014). The density and reproductive success of breeding pairs, however, has been found to vary with the slope of the intertidal area that influences foraging availability (Andres, 1998; Hazlitt, 2001). Although habitat use is expected to change as reproductive duties conclude, habitat studies outside the breeding season are extremely limited (Hartwick & Blaylock, 1979).

Here, we deploy satellite tags on black oystercatchers in four regions of British Columbia, Canada, and use tracking data to examine habitat use and habitat selection decisions across seasonal, diel, and tidal cycles. We describe the shoreline within each region, examine habitat use, and investigate temporal variation in the habitat selection decisions of black oystercatchers. We expected that black oystercatchers would exhibit preferences for shoreline habitat where predation risk was reduced during the breeding season, at night and during high tides, and would exhibit preferences for shoreline habitat with greater foraging rewards during the nonbreeding season, the day and during low tides. We also evaluate whether detailed examination of variation in the frequency that individuals revisit key sites within their home range yield additional insights into the habitat preferences of black oystercatchers.

## 2 MATERIALS AND METHODS

### 2.1 Study species and area

**FIGURE 1:**
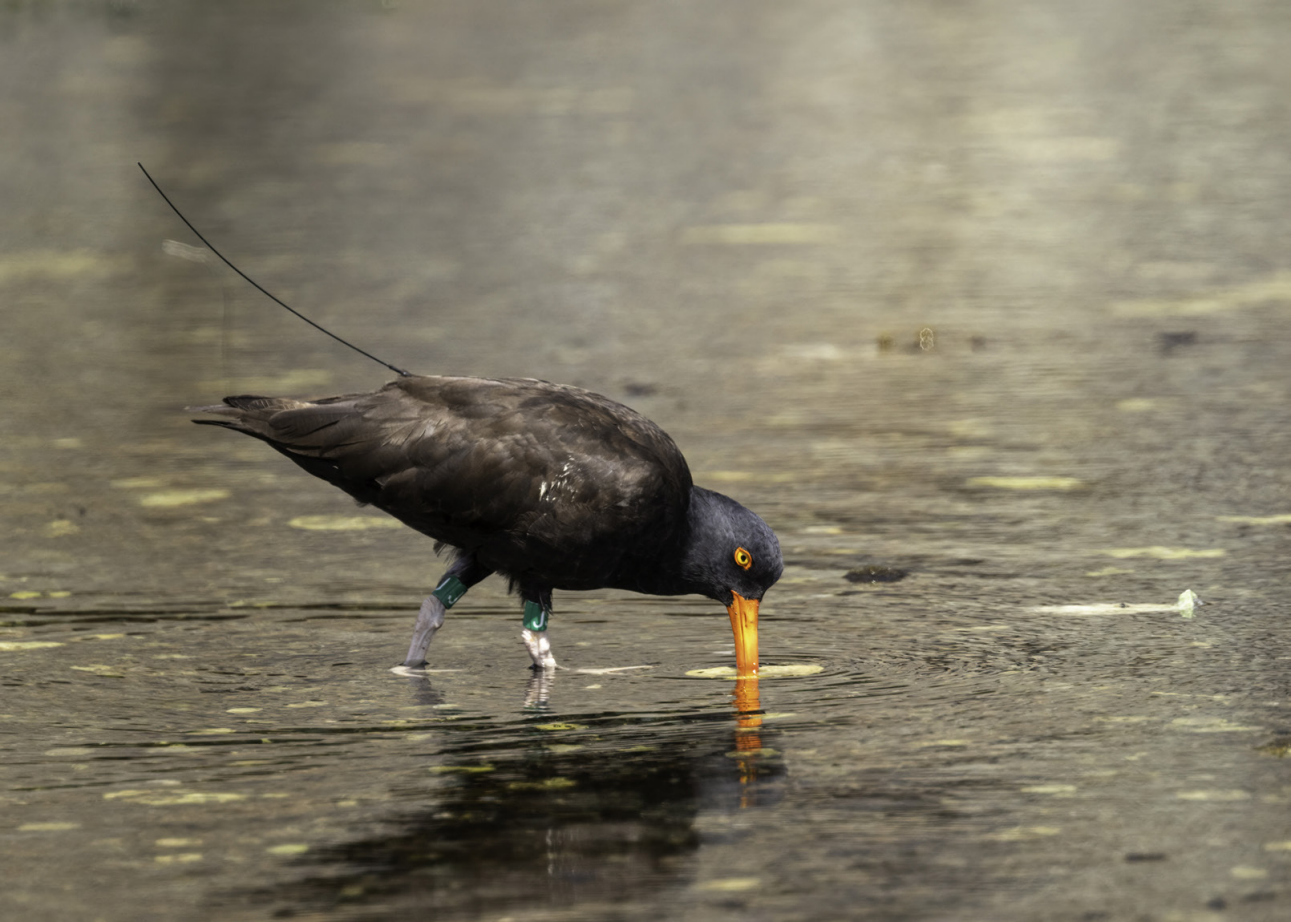
Tracked black oystercatcher photographed August 3, 2020, in Nanaimo, British Columbia, by Pius Sullivan. This individual was captured on March 3, 2019, and fit with a solar‐powered Argos satellite transmitter (9.5 g, PTT‐100, Microwave Technology) with a leg‐loop style harness.

The black oystercatcher (*Haematopus bachmani*; Figure 1) is a relatively large (500–700 g) conspicuous shorebird found along the Pacific coast of North America. Black oystercatchers feed exclusively on intertidal macro‐invertebrates, particularly mussels, limpets, and chitons (Hartwick, 1973; Hazlitt & Gaston, 2002) and are thought to be a sensitive indicator of the overall health of the rocky intertidal community (Tessler et al., 2014). Monitoring suggests that the global population is stable (global population estimates range from 12,500 to 17,500; Bennett, 2019; Tessler et al., 2014; Weinstein et al., 2014), although they remain a species of concern because of their restricted habitat and sensitivity to human disturbance (Burger, 1986; Goss‐Custard & Verboven, 1993; Peters & Otis, 2006; Yasué, 2006). Predation of adult black oystercatchers is not well documented but raptors are the suspected predators (e.g., peregrine falcon, bald eagle; Tessler et al., 2014). Predation of eggs and chicks is significant enough that nests are rarely found on accessible mainland shores (Andres & Falxa, 2020). Documented nest predators include mammals (e.g., mustelids, foxes, and bears) and birds (e.g., corvids and gulls; Morse et al., 2006; Spiegel et al., 2012).

**FIGURE 2:**
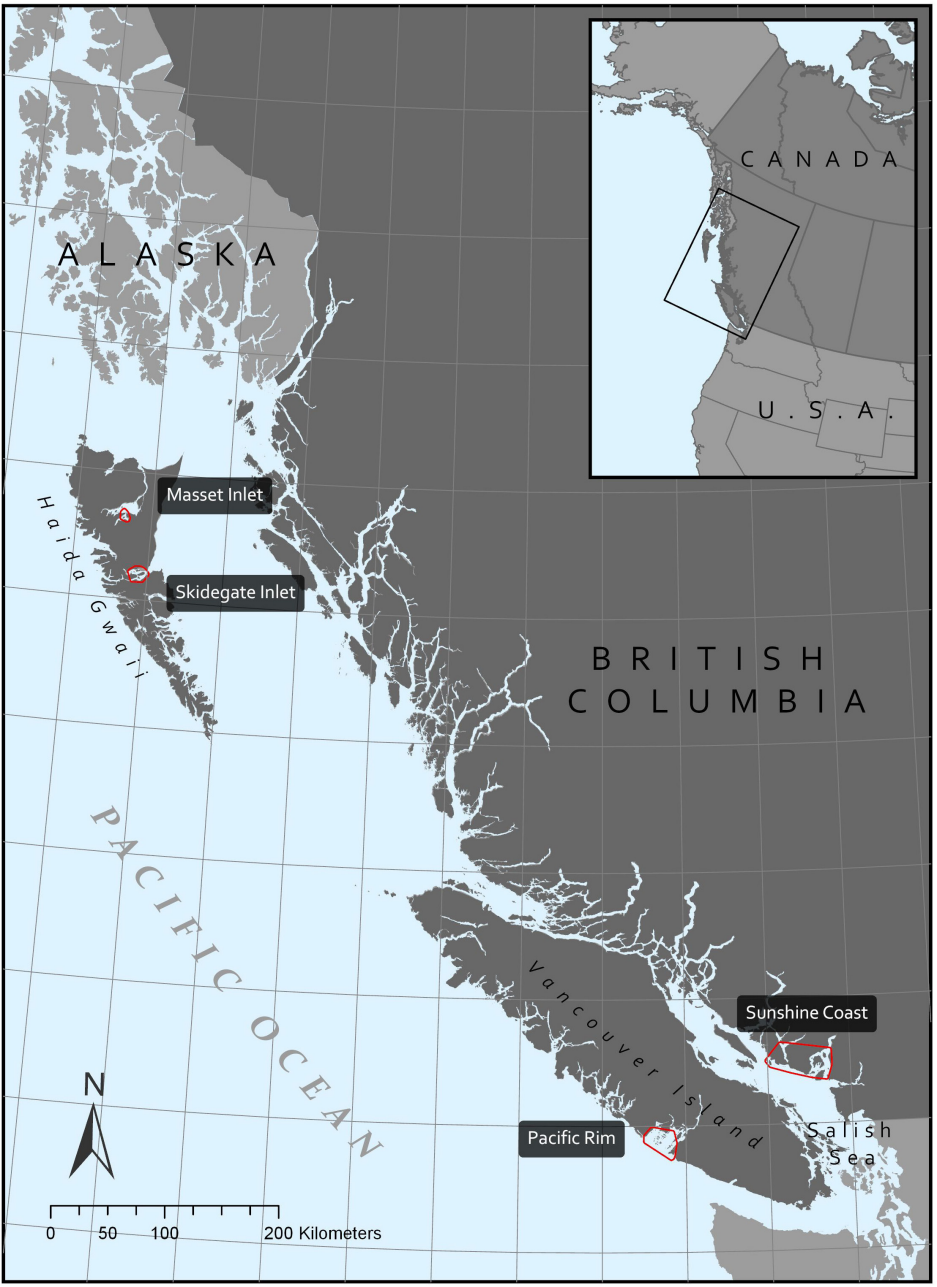
Four regions in British Columbia, Canada, where tracked black oystercatchers were studied. Red outlines represent the 95% MCP study areas for combined satellite tracking data. Gridlines mark each degree of latitude and longitude. Coordinate System: NAD 1983. Projection: BC Environment Albers.

We studied black oystercatchers at four regions characteristic of coastal British Columbia (hereafter B.C.; Figure 2): the Sunshine Coast, the Pacific Rim National Park Reserve area on the west coast of Vancouver Island (hereafter Pacific Rim), Skidegate Inlet and Masset Inlet on the archipelago of Haida Gwaii. The Sunshine Coast region is located within the Salish Sea, a relatively protected body of water with considerable freshwater and sediment discharge from several major rivers (Gilkeson et al., 2006). The Pacific Rim region has an exposed coastline with nutrient‐rich nearshore waters (Parks Canada Agency, 2021). The Haida Gwaii archipelago, located on the northern B.C. coast, is rugged and exposed although Skidegate Inlet and Masset inlet are sheltered from the elements. The B.C. coast experiences relatively mild winters (i.e., rarely freezing) making the nearshore habitats attractive to waterbirds, especially during the winter months (Ethier et al., 2020). Pacific Rim, Skidegate Inlet, and Masset Inlet remain sparsely populated by rural communities. In contrast, the broader area surrounding the Sunshine Coast is densely populated and impacted by human pressures (Sobocinski, 2021; Stocks & Vandeborne, 2017).

### 2.2 Field methods

We captured black oystercatchers using noose‐mats, decoys (Mad River Decoys), and an audio lure (Foxpro Inferno). Black oystercatchers were banded with a USGS stainless steel band on the tarsus and a pair of green alpha‐numeric plastic bands on the tibia (Haggie Engraving, Millington, MD). We assigned individuals a probable sex using the eye‐fleck technique described by Guzzetti et al. (2008), where females show a moderate to distinct eye‐fleck and males show little to no eye‐fleck. We classified individuals as subadult (second year) or adult (after second year) based on the brightness of the eye and bill (Andres & Falxa, 2020).

We deployed 20 solar‐powered Argos satellite transmitters (9.5 g, PTT‐100, Microwave Technology) on adult black oystercatchers captured between February 28 and March 28, 2019 (Sunshine Coast *n* = 4, Pacific Rim *n* = 6, Skidegate Inlet *n* = 6, Masset Inlet = 4). Transmitters, glued to a 3 mm neoprene patch to prevent abrasion, were mounted on the synsacrum of the birds using a leg‐loop harness (Mallory & Gilbert, 2008) made from Teflon ribbon tubing (Bally Ribbon Mills). We reinforced harnesses with nylon trammel‐line because oystercatchers can rapidly remove harnesses made with Teflon ribbon alone (Johnson et al., 2010; Loring et al., 2017). Transmitter units (3.9 x 1.8 x 1.3 cm) and harness had a combined mass of 10.5 g representing, on average, 1.7% (range: 1.5%–2.0%) of the bird's mass (body mass ± SD: 622 ± 44 g). Satellite transmitters were programmed to transmit 1 h ON and 48 h OFF when they had suitable solar charge and switched off when they did not have suitable charge.

We attempted to observe tagged birds in the field between April and July 2019 to determine whether individuals were actively defending breeding territories. We classified individuals as “breeding” if they were observed as part of a territorial pair in suitable nesting habitat, or with an active nest or young. Birds were classified as “non‐breeding” if they were unpaired, not exhibiting territorial behavior, and/or observed in unsuitable nesting habitat during the breeding season.

### 2.3 Satellite tracking data

During the initial screening of the tracking data, we retained locations assigned an Argos accuracy class of 3 (radius of error: <250 m), 2 (radius of error: 250–500 m), or 1 (radius of error: 500–1500 m) and discarded data assigned an accuracy class of 0 (radius of error: >1500 m), A, B, and Z (no accuracy estimate). We also discarded data from one individual from Skidegate Inlet that was not tracked during both the breeding and nonbreeding season. The screened dataset included 17,954 locations collected from 19 individuals tracked for the 12‐month period (*n* = 562–1256 locations per individual). We observed significant seasonal bias in the raw data which is somewhat typical for solar‐powered tracking devices at mid‐to‐high latitudes (Silva et al., 2017), with the majority of the locations occurring during the summer months and the daytime. We compared (1) the probability that tracked individuals survived for a year with the estimated annual apparent survival of black oystercatchers in B.C. (0.90 ± 0.03 SE; P. Clarkson & Y. Zharikov unpubl. data reported in Tessler et al., 2014) and (2) the proportion of tracked individuals and the proportion of individuals color‐banded at the same sites in 2019 that were visually resighted more than a year after tagging/banding. Post hoc contrasts were conducted with proportions and standard errors estimated from the proportions and estimates ± SE of apparent annual survival using Program Contrast (Hines & Sauer, 1989; “https://www.mbr‐pwrc.usgs.gov/software/contrast.html”).

We identified whether locations were collected during the breeding or nonbreeding season, day or night, and high or low tide. We defined the “breeding season” as the 4 months from April to July when paired individuals are most likely defending a territory, incubating eggs, or caring for young (Andres & Falxa, 2020), and the “non‐breeding season” as the 8 months from August to March when individuals are less likely to be restricted to a central location. We defined “daytime” as the hours between sunrise and sunset, and “nighttime” as the hours between sunset and sunrise. We determined sunrise/sunset times for each date and location using the R package “suncalc” (Thieurmel & Elmarhraoui, 2019). We classified “high” and “low” tides as the highest and lowest 50% of tide height predictions, by tide station and week of the year. We obtained hourly tide height predictions from 15 tide stations (“http://tbone.biol.sc.edu/tide”) and linked stations to each Argos location based on their proximity and the geography of the coastline.

To reduce bias associated with unequal sampling across individuals and time of year (see above), we retained four locations per week per individual. We selected a ratio of three daytime to one nighttime location to preserve the original proportion of day to night locations in the dataset, randomly selecting data from the most accurate Argos class available in each week (time intervals between locations averaged 29 h, range = 1–127). The resulting reduced dataset included 2681 locations, with 19 individuals contributing an average of 141 data points (range = 117–161). Time intervals between locations averaged 57 h apart (range: 1–768). There were time gaps in the data obtained from individuals during winter months; however, location data were split evenly between the breeding and nonbreeding periods and high and low tides. The majority of locations in the reduced dataset were from the most accurate class (86% class 3 locations, 10% class 2 locations, and 4% class 1 locations).

### 2.4 Description of shoreline

**FIGURE 3:**
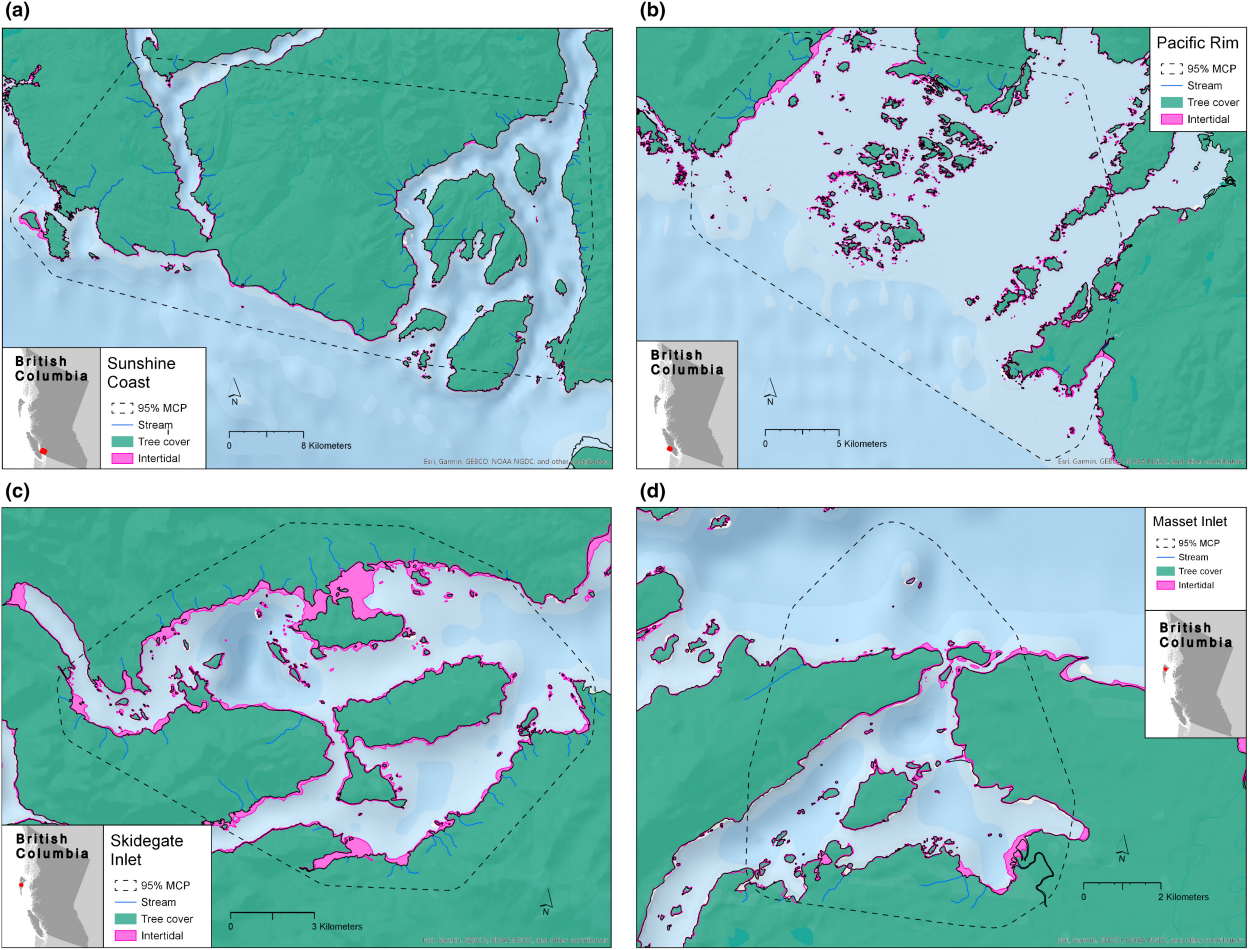
Maps indicating the four 95% MCP study areas where tracked black oystercatchers with overlapping home ranges spent the year: (a) Sunshine Coast *n* = 4, (b) Pacific Rim *n* = 5, (c) Skidegate Inlet *n* = 5, (d) Masset Inlet *n* = 4.

**FIGURE A1:**
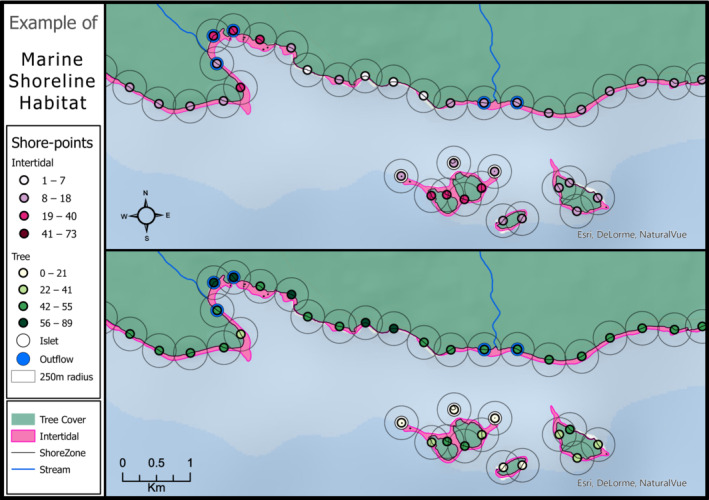
Visual aid for the description of available marine shoreline habitat and shore‐points. The “Intertidal” variable represents the percent intertidal area within a 250 m radius of a shore‐point. The “Tree” variable represents the percent tree cover within a 250 m radius of a shore‐point. “Islet” shore‐points occur on islands with an area less than 1.5 ha, and “Outflow” shore‐points occur within 500 m of a freshwater stream mouth.

We described the marine shoreline habitat within region‐specific 95% Minimum Convex Polygons (MCP; Figure 3), generated using all locations in the reduced dataset of individuals with overlapping home ranges (Sunshine Coast *n* = 4, Pacific Rim *n* = 5, Skidegate Inlet *n* = 5, Masset Inlet = 4). Within MCP study areas, we created representative habitat units, hereafter “shore‐points,” at a resolution matching the satellite tracking data (approximately 250 m). To do this, we generated point locations every 500 m along the shoreline in ArcGIS Pro (version 3.2.0); the straight‐line distance between shore‐points averaged 290 m (range: 65–2110 m; see Figure A1). We used the B.C. ShoreZone polyline dataset to represent shoreline (Howes et al., 1994). We manually created additional shore‐points on islets/islands, or groups of islets/islands separated by <250 m, that were not captured by the automated tool because their shoreline was less than 500 m in length. This resulted in a total of 2125 shore‐points (Sunshine Coast *n* = 834, Pacific Rim *n* = 776, Skidegate Inlet *n* = 362, Masset Inlet = 153).

Shoreline habitat use by shorebirds can be influenced by prey availability, perceived risk of predation and human disturbance (Burger, 1986; Goss‐Custard & Verboven, 1993; Hope et al., 2020; Johnston‐González & Abril, 2019; Peters & Otis, 2006; Pomeroy, 2006; Schwemmer & Garthe, 2011; Sprague et al., 2008; Yasué, 2006). We described shore‐points using seven variables associated with either prey availability and predation risk (Table 1); variables associated with human disturbance (e.g., distance to roads and proportion of urban development) were considered but not examined because there was little to no urban development within three of the four regions.

**TABLE 1:** Variables hypothesized to influence black oystercatcher use of marine shoreline.

Function	Name	Description	Data type	Source	Retained
Habitat selection across seasonal, diel and tidal cycles					
Foraging opportunity	Intertidal_250_	Percent intertidal area within a 250 m radius of shore‐point	Continuous	Canadian Hydrological Service (2020)	Yes
	Substrate	ShoreZone coastal class (reclassified)	Categorical (6 classes)	Howes et al. (1994)	No
	Exposure	ShoreZone wave exposure estimate	Ordinal	Howes et al. (1994)	No
	Outflow	Occurrence of a freshwater outflow within 500 m of shore‐point	Binary	BC Freshwater Atlas (2020)	Yes
	Southness	Aspect of shoreline (rescaled to 0–180 degrees)	Continuous	Ware (2021)	No
Safety	Tree_250_	Percent tree cover within a 250 m radius of shore‐point	Continuous	Ware (2021)	Yes
	Islet	Small rocky island lacking trees (area < 1.5 ha)	Binary	Ware (2021)	Yes
Repeated use of islets within home ranges					
Foraging opportunity	Intertidal_1000_	Percent intertidal area within a 1000 m radius of islet	Continuous	Canadian Hydrological Service (2020)	Yes
	OutflowDist	Distance to nearest freshwater outflow (km)	Continuous	BC Freshwater Atlas (2020)	Yes
Safety	Tree_1000_	Percent tree cover within 1000 m radius of islet	Continuous	Ware (2021)	Yes
	IsletArea	Area of islet (hectares)	Continuous	Ware (2021)	Yes
Repeated use of outflows within home ranges					
Foraging opportunity	Intertidal_1000_	Percent intertidal area within a 1000 m radius of outflow	Continuous	Canadian Hydrological Service (2020)	Yes
	Substrate	ShoreZone coastal class (reclassified)	Categorical (6 classes)	Howes et al. (1994)	Yes
	Southness	Aspect of shoreline (rescaled to 0–180 degrees)	Continuous	Ware (2021)	Yes
	StreamOrder	Order of stream at shoreline intersection (2–5)	Ordinal	BC Freshwater Atlas (2020)	Yes
Safety	Tree_1000_	Percent tree cover within a 1000 m radius of outflow	Continuous	Ware, 2021)	Yes

The availability and abundance of marine invertebrates in the intertidal zone can be influenced by several physical characteristics of the shoreline, including substrate, wave exposure, slope, aspect, and nutrient supply (Kaiser et al., 2011). We assigned shore‐points as substrate class (“bedrock,” “mixed‐rock,” “gravel,” “sand,” “mudflat,” and “man‐made”) and a wave exposure class (“very protected,” “protected,” “semi‐protected,” “semi‐exposed,” “exposed,” and “very exposed”) using the ShoreZone dataset for B.C. (Howes et al., 1994). ShoreZone uses 34 coastal classes, and these were combined into six broader substrate categories following Dalgarno et al., 2017. We estimated the percent cover of intertidal habitat within a 250 m radius of shore‐points (Intertidal_250_) using a spatial layer describing the intertidal area exposed during “Lowest Normal Tide” (“https://tides.gc.ca/en/vertical‐datum‐chart‐references”). We created this layer by converting the Canadian Hydrographic Service “high water mark” and “low water mark” polylines (“https://data.gov.bc.ca/”) into polygons representing the exposed intertidal area. We determined the aspect of each shore‐point using 100 x 100 m resolution Digital Elevation Models for each location (“https://maps.canada.ca/”). We then converted circular aspect values (0–360) to a southness score (0–180) since south‐facing slopes receive more solar energy than north‐facing areas. Finally, we categorized shore‐points as being associated with a freshwater outflow (hereafter outflows; Outflow: yes/no) if they were within 500 m of a permanent freshwater stream (stream orders 2–5). We chose 500 m because a freshwater source influences the marine environment several hundred meters from a rivers mouth and varies by stream size and season (Tallis, 2009).

The risk of nest depredation by mammals and birds likely explains why black oystercatchers prefer to nest on islets and coastline some distance from trees (Dalgarno et al., 2017; Hazlitt & Gaston, 2002; Weinstein et al., 2014). Shorebird foraging habitat selection is also influenced by predation risk (Hope et al., 2011; Ydenberg et al., 2004); trees/vegetation are perceived as being dangerous because they can conceal raptors (Johnston‐González & Abril, 2019; Pomeroy, 2006; Sprague et al., 2008). We classified shore‐points as islets (Islet: yes/no) if a shore‐point was located on a small island with an area less than 1.5 hectares. We chose the threshold of 1.5 hectares because larger islands supported trees. We estimated the percent cover of tree cover within a 250 m radius of shore‐points (Tree_250_) using a manually digitized spatial layer describing the tree cover at each site using satellite imagery (ESRI Basemap Imagery) at a 1:5000 scale.

### 2.5 Habitat selection across seasonal, diel, and tidal cycles

For each region, we determined whether shore‐points were used by black oystercatchers during the breeding and nonbreeding season, day and night, and high and low daytime tides. A shore‐point was “used” if satellite tracking location from one or more individuals occurred within 250 m of a shore‐point. We used six paired generalized linear mixed models (GLMM) to examine whether habitat characteristics associated with foraging opportunity and safety influenced the use of a shore‐point during the two seasons, two diel and two tidal periods. GLMMs included region as a random term and were fitted using a binomial distribution and logit link with the R package “glmmTMB” (Brooks et al., 2017). We reduced the spatial autocorrelation in the models by comparing used shore‐points (*n* = 388) with a randomly selected subset (50%) of available shore‐points (*n* = 1063). However, inspection of the model residuals and correlogram showed that significant spatial autocorrelation remained for shore‐points within 500 m (Morans I > 2.0 for all models). We therefore generated and included a spatial autocovariate term in each model that reduced spatial autocorrelation in the model residuals to negligible levels (Fortin et al., 2002).

**FIGURE A2:**
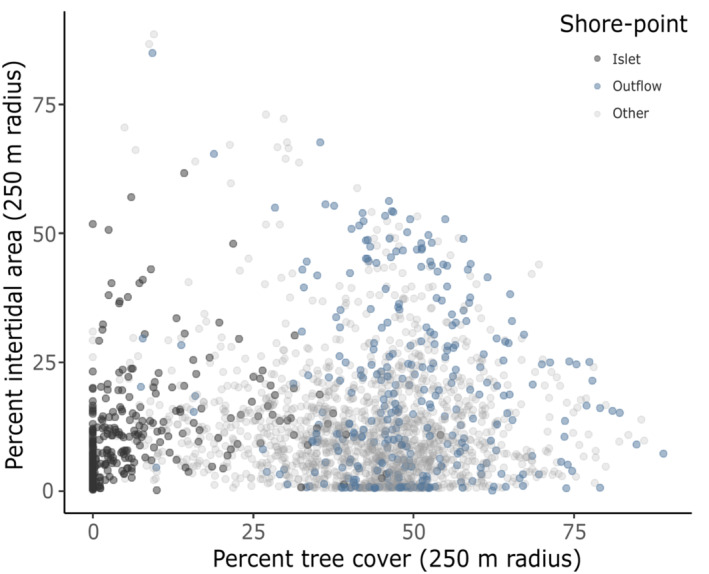
Each data point represents a shore‐point included in the analysis evaluating habitat use by black oystercatchers over seasonal, diel, and tidal cycles (*n* = 1063). Shore‐points are locations generated every 500 m along the shoreline that represent habitat units and average 290 m apart depending on the ruggedness of the coastline (range: 65–2110 m). Percent intertidal area within a 250 m radius of shore‐points (range: 0–89, mean ± SD: 14.0 ± 13.2) is plotted against percent tree cover within the same radius (range: 0–86, mean ± SD: 36.4 ± 19.9). 14.7% shore‐points are classified as islets and 15.3% of shore‐points are classified as occurring near a freshwater outflow.

Prior to conducting our analyses, we examined variation and collinearity of habitat variables. We excluded the exposure variable because it did not contain enough variation within regions to drive habitat use at a 250 m scale. All shore‐points identified as “islets” were of the same substrate class and contained null aspect values, so we excluded Southness and Substrate here but re‐examined them in the third analysis (see Section 2.7 below “Characterizing variation in islets and outflows”). We assessed the relationship between the two binary variables, Islet and Outflow, and the remaining descriptive variables. Islets were associated with low percent cover of trees and intertidal, and outflows tended to be associated with larger percent cover of trees and intertidal (Figure A2); however, variation inflation factors remained low (VIF < 3), suggesting that the inclusion of all four variables was not problematic (Zuur et al., 2010). The final models included the two binary variables, Outflow (15.3% of shore‐points) and Islet (14.7% of shore‐points) as well as two numerical variables, Intertidal_250_ (range: 0–87, mean ± SD: 14 ± 13) and Tree_250_ (range: 0–86, mean ± SD: 36 ± 20).

### 2.6 Description of individual home ranges

Methods for estimating home range of animals constrained to linear features (e.g., canyons, rivers, and coastlines) are prone to overestimation of actual space use (Slaght et al., 2013; Tarjan & Tinker, 2016). To allow comparison with other studies and species, we estimated the home range of the 19 individuals tracked for the entire year by generating 95% minimum convex polygons (MCP) using the R package “adehabitathr” (Calenge, 2006). We also estimated the length of shoreline within each home range that was used by generating a 250 m buffer around the point locations and examined the proportion of the total shoreline within each home range that was used by each bird.

### 2.7 Characterizing variation in islets and outflows

We identified 131 islets (Sunshine Coast *n* = 14, Pacific Rim *n* = 75, Skidegate Inlet *n* = 25, Masset Inlet *n* = 17) and 39 outflows (Sunshine Coast *n* = 13, Pacific Rim *n* = 6, Skidegate Inlet *n* = 15, Masset Inlet *n* = 5) visited by a tracked black oystercatcher at least once during the annual cycle. We described islets using two variables linked to foraging opportunities in the vicinity of the islet (percent cover intertidal area within a 1000 m radius centered on the islet, distance to nearest outflow) and two variables linked to the perceived risk of danger (islet size and percent tree cover within a 1000 m radius centered on the islet; Table 1). We described the outflows using four variables linked to prey availability (percent intertidal area within a 1000 m radius centered on the stream output, substrate type, aspect, and stream order) and one variable linked to the perceived risk of predation (percent tree cover within a 1000 m radius; Table 1). For each islet and outflow visited by an individual, we also calculated the distance (kilometers) from the islet or outflow to the center of an individual's core home range (50% MCP). We quantified the percent cover of intertidal area and tree cover surrounding islets and outflows within a 1000 m radius, rather than a 250 m radius, because islets and outflows were spatially segregated, and our goal was to describe the foraging opportunities and perceived predation risk around these features at a scale relevant to an individual oystercatcher rather than describe each segment of the shoreline at a scale relevant to the accuracy of the tracking data. We calculated the distance from islets to the nearest freshwater outlet using the Euclidian distance from the center of the islet to the closest perennial stream. We estimated islet size using the area that was above the mean high tide line. We determined the stream order at the point the stream intersected the intertidal zone. Stream order is a commonly used ordinal metric to group stream segments by size and similar hydraulic properties. Stream locations and attributes were obtained from the B.C. Freshwater Atlas (“https://catalogue.data.gov.bc.ca/”). Other metrics were calculated as described previously.

### 2.8 Repeated use of islets and outflows within home ranges

We determined the number of times individuals were tracked to islets and outflows using the reduced satellite tracking dataset and the “recurse” package in R (Bracis et al., 2018). Islets and outflows were identified by a point location (coordinates), and individuals were assumed to have visited that feature if a tracking location fell within 500 m of the point location. A 500 m radius was used instead of 250 m because the intertidal flats associated with larger streams tended to be several hundred meters from the stream mouth proper. Visits were not considered separate events unless the individual was tracked elsewhere prior to returning to the islet or outflow. We used visits as a metric for repeated use instead of the number of tracked locations because it helped reduce the bias associated with breeding sites and we were interested in sites that were returned to often, rather than sites used for extended periods of time (Bracis et al., 2018; Wittemyer et al., 2019).

We developed two candidate sets and used GLMMs to investigate how the characteristics of islets and outflows within the home ranges of individual oystercatchers influenced the intensity of their use. All models in both candidate sets included individual and region as a random term and were fitted using a negative binomial distribution for overdispersed count data (Brooks et al., 2017). The candidate set examining the intensity with which individuals used different islets included univariate and multivariate models with all combinations of the four metrics used to describe islets (Tree_1000_, IsletArea, Intertidal_1000_, OutflowDist; *n* = 16 models). All models in this set included a variable (CentroidDist + CentroidDist^2^), which controlled for the nonlinear distance from the center of an individual's core range to each islet visited, and an autocovariate that corrected the spatial autocorrelation in the model residuals. The candidate set examining the intensity with which individuals used freshwater outflows included all univariate models and multivariate models with combinations of two and three of the six metrics used to describe outflows (Tree_1000_, Intertidal_1000_, Substrate, StreamOrder, Southness; *n* = 26 models). Models with more terms failed to converge. Models in this set did not include CentroidDist or a spatial autocovariate. Preliminary analyses indicated that the intensity with which individuals visited outflows was not influenced by their proximity to the center of an individual's core range and there was no evidence for spatial autocorrelation in the model residuals (Morans I < 2.0).

We ranked models in each candidate set by Akaike's information criterion (AICc) and Akaike weights (w*i*) (Burnham & Anderson, 2004); models with the lowest AIC values were identified as the most parsimonious. We calculated ΔAIC as the difference in AIC between each model and the top model and reported % model deviance, marginal and conditional *R* ^2^. We used the R package “glmmTMB” to build the models (Brooks et al., 2017) and the package “performance” to estimate marginal and conditional *R* ^2^ (Nakagawa & Schielzeth, 2013). We validated the assumptions of the models by inspecting the observed model residuals against the expected with the package “DHARMa” (Hartig, 2020). All statistical analyses were conducted using R Studio 3.6.1 (R Development Core Team 2015).

## 3 RESULTS

### 3.1 Tracking black oystercatchers

**FIGURE A3:**
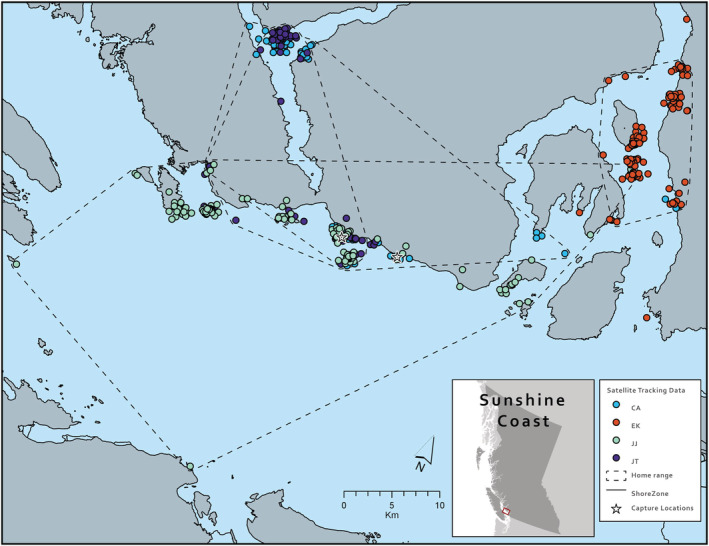
Map of the Sunshine Coast region with processed satellite tracking data from four black oystercatchers over one annual cycle (March 2019–2020) and associated home ranges (95% MCP).

**FIGURE A4:**
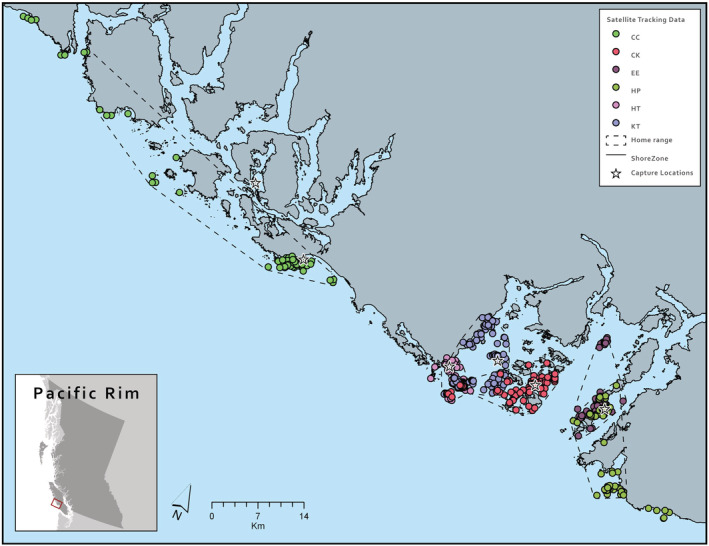
Map of the Pacific Rim region with processed satellite tracking data from six black oystercatchers over one annual cycle (March 2019–2020) and associated home ranges (95% MCP). The individual with band code “CC” was excluded from the habitat selection analysis.

**FIGURE A5:**
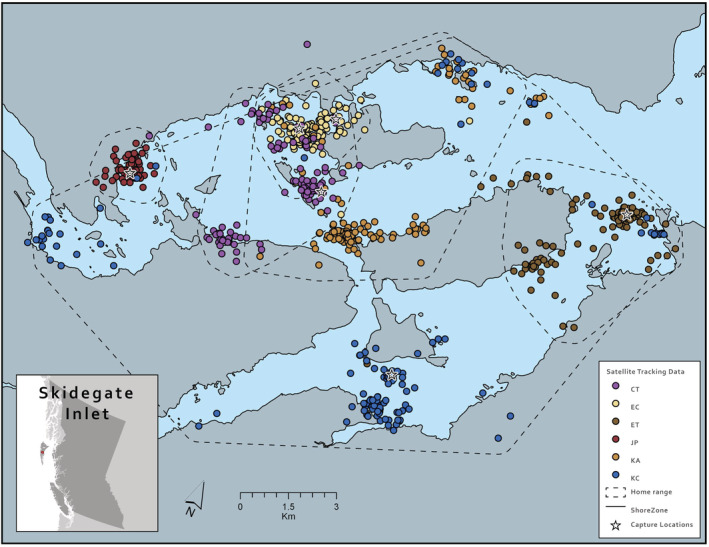
Map of the Skidegate Inlet region with processed satellite tracking data from six black oystercatchers over one annual cycle (March 2019–2020) and associated home ranges (95% MCP). The individual with band code “JP” only represents the first 6 months of tracking.

**FIGURE A6:**
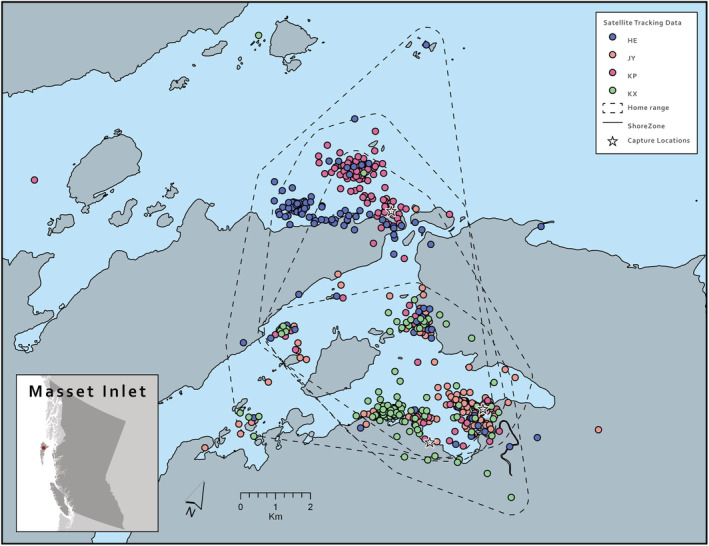
Map of the Masset Inlet region with processed satellite tracking data from four black oystercatchers over one annual cycle (March 2019–2020) and associated home ranges (95% MCP).

We tracked the movements of 20 black oystercatchers for 6–12 months. The black oystercatchers that carried transmitters all were nonmigratory and generally remained within the region they were captured (Figures A1, A2, A3, A4, A5, A6). One transmitter failed after 6 months and was recovered near its nesting site under the perch of a bald eagle. Nineteen individuals were tracked for approximately 1 year, and eight continued to transmit into the following year. The probability that tracked individuals survived for 1 year (0.95 ± 0.049 SE) did not differ from the estimated annual survival of black oystercatchers in B.C. (0.90 ± 0.03 SE; P. Clarkson & Y. Zharikov unpubl. data reported in Tessler et al., 2014; chi‐square = 0.76, df = 1, *p* = .38). The probability that tracked individuals were visually resighted during monitoring surveys in subsequent years also did not differ from the probability that birds color‐banded at the same sites in 2019 (*n* = 23) were resighted (tracked: 0.650 ± 0.107, color‐banded only: 0.565 ± 0.103, chi‐square = 0.32, df = 1, *p* = .57). Nine individuals with failed tags were later observed alive without transmitters and harnesses (see corresponding thesis for more detail; Ware, 2021).

### 3.2 Habitat selection across seasonal, diel, and tidal cycles

**FIGURE 4:**
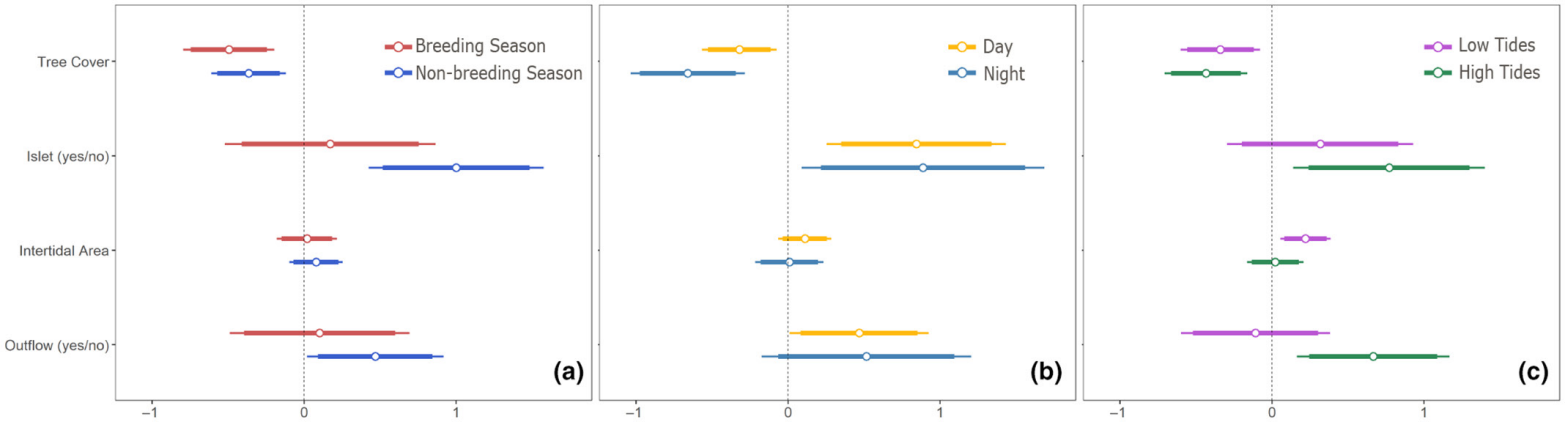
Standardized effects plot indicating the influence of four habitat variables on shoreline use by black oystercatchers during the (a) breeding season and nonbreeding season, (b) day and night, (c) low and high tides. A shore‐point was considered “used” by an oystercatcher if a satellite location from one or more individuals occurred within 250 m during the focal time period. “Used” shore‐points were compared with a random subset of “available” shore‐points using generalized linear mixed models with region as a random term. The units are standard deviation.

Tracked black oystercatchers used 11%–39% of the shore‐points that described the available marine shoreline (Sunshine Coast = 11%, Pacific Rim = 15%, Skidegate Inlet = 32%, Masset Inlet = 39%). Black oystercatchers used islets more than expected based on their availability, during the nonbreeding season (*z* = 3.7, *p* < .001), but this pattern was not observed in the breeding season (Figure 4a; *z* = 0.9, *p* = .36). Islets were also used more than expected during the day (*z* = 3.3, *p* < .001) and night (*z* = 2.1, *p* < .05; Figure 4b), and during periods of high tides (*z* = 2.8, *p* < .05; Figure 4c) when access to the intertidal zone was restricted. Black oystercatchers used outflows more than expected during the nonbreeding season (*z* = 2.5, *p* < .05) but not during the breeding season (*z* = 0.3, *p* = .7). Outflows were also used more than expected during daylight hours (*z* = 2.3, *p* < .05) but not during the night (*z* = 1.4, *p* = .2). Outflows were used more than expected during high tides (*z* = 3.0, *p* < .05) and not low tides (*z* = −0.4, *p* = .7) which was counter to expectation.

Black oystercatcher preferences for shoreline with greater intertidal area were not apparent when assessed at a seasonal scale; populations did not use shoreline with more intertidal habitat during either the nonbreeding or breeding season (nonbreeding: *z* = 1.8, *p* = .07; breeding: *z* = 1.0, *p* = .34). Shoreline with greater intertidal areas was used more than expected during daylight hours (day: *z* = 1.9, *p* = .05; night: *z* = 1.0, *p* = .31) and during low tides (low tide: *z* = 3.0, *p* < .05; high tide: *z* = 0.8, *p* = .42).

Black oystercatchers avoided shoreline with greater tree cover during all time periods. Avoidance of tree cover was stronger during the breeding season relative to the nonbreeding season, stronger at night relative to the day, and stronger during high tides relative to low tides. The GLMMs that evaluated the importance of habitat characteristics on shoreline use by black oystercatchers explained 43%–58% of the variation in the data (marginal *R* ^2^ = 0.43–0.58, percent deviance = 3%–24%).

### 3.3 Description of individual home range

We estimated home range size and associated linear shoreline of 19 black oystercatchers (Sunshine Coast = 4, Pacific Rim = 6, Skidegate Inlet = 5, Masset Inlet = 4). Black oystercatcher home range size over the course of 1 year encompassed an area of, on average, 153 km^2^, but there was enormous variation among individuals (95% MCP range: 5–1193 km^2^). Individuals on the Sunshine Coast had larger home ranges than those residing at the other three sites (mean 95% MCP ± SD: Sunshine Coast = 501 ± 483, Pacific Rim = 92 ± 91, Skidegate Inlet = 44 ± 61, Masset Inlet = 25 ± 13; controlling for breeding status and sex, *F*_3,11_ = 6.5, *p* < .01). Black oystercatchers that were confirmed as breeding (*n* = 7) also tended to have smaller home ranges than those that were later confirmed as not breeding (*n* = 5), or whose breeding status was unknown (*n* = 3; mean ± SD; breeding = 56 ± 77, nonbreeding = 282 + 510, unknown status = 159 ± 160, *F*_2,11_ = 2.4, *p* = .14; breeding‐nonbreeding contrast: *t* = −2.6, *p* = .02). The sex of an individual had no detectable effect on the size of their 95% MCP (female = 298 ± 502, *n* = 5; male = 112 ± 128, *n* = 5; unknown sex = 97 ± 142, *n* = 9; *F*_2,11_ = 4.0, *p* = .05; female–male contrast: *t* = 0.58, *p* = .57). Black oystercatcher home ranges contained, on average, 46 km of shoreline (range: 12–156 km) with larger home ranges containing more linear shoreline habitat (*r*_p_ = 0.92, *p* < .01). Black oystercatchers used, on average, 10.4 km or 33% of the shoreline within their home range; however, the accuracy of the satellite locations (200–1500 m) made this difficult to estimate with precision. All birds used a similar length of shoreline (range: 6.7–13.9 km) as individuals with larger home ranges used a smaller proportion of the shoreline available than those with smaller home ranges (*r*_p_ = −0.60, *p* < .01).

### 3.4 Repeated use of key habitat features within home ranges

Black oystercatchers made extensive use of islets and outflows within their home ranges. Due to the temporal resolution of the tracking data (maximum of four locations per week), visits to key shoreline features are relative and represent a proportion of actual visits. Individuals visited, on average, 11 islets (range: 3–29, *n* = 19) but returned to some islets up to 29 different times while using other islets only once. Individuals visited an average of four freshwater outflows (range: 1–8, *n* = 16) in 1 year, from one to 10 times. Islets were visited more often if they were closer to the center of an individual's core home range area as a nonlinear effect of distance (ΔAICc: Null model = 42, CentroidDist = 19, CentroidDist + CentroidDist^2^ = 15) but this did not influence the use of outflows (ΔAICc: Null model = 20, CentroidDist = 21, CentroidDist + CentroidDist^2^ = 24).

#### 3.4.1 Islets

**FIGURE 5:**
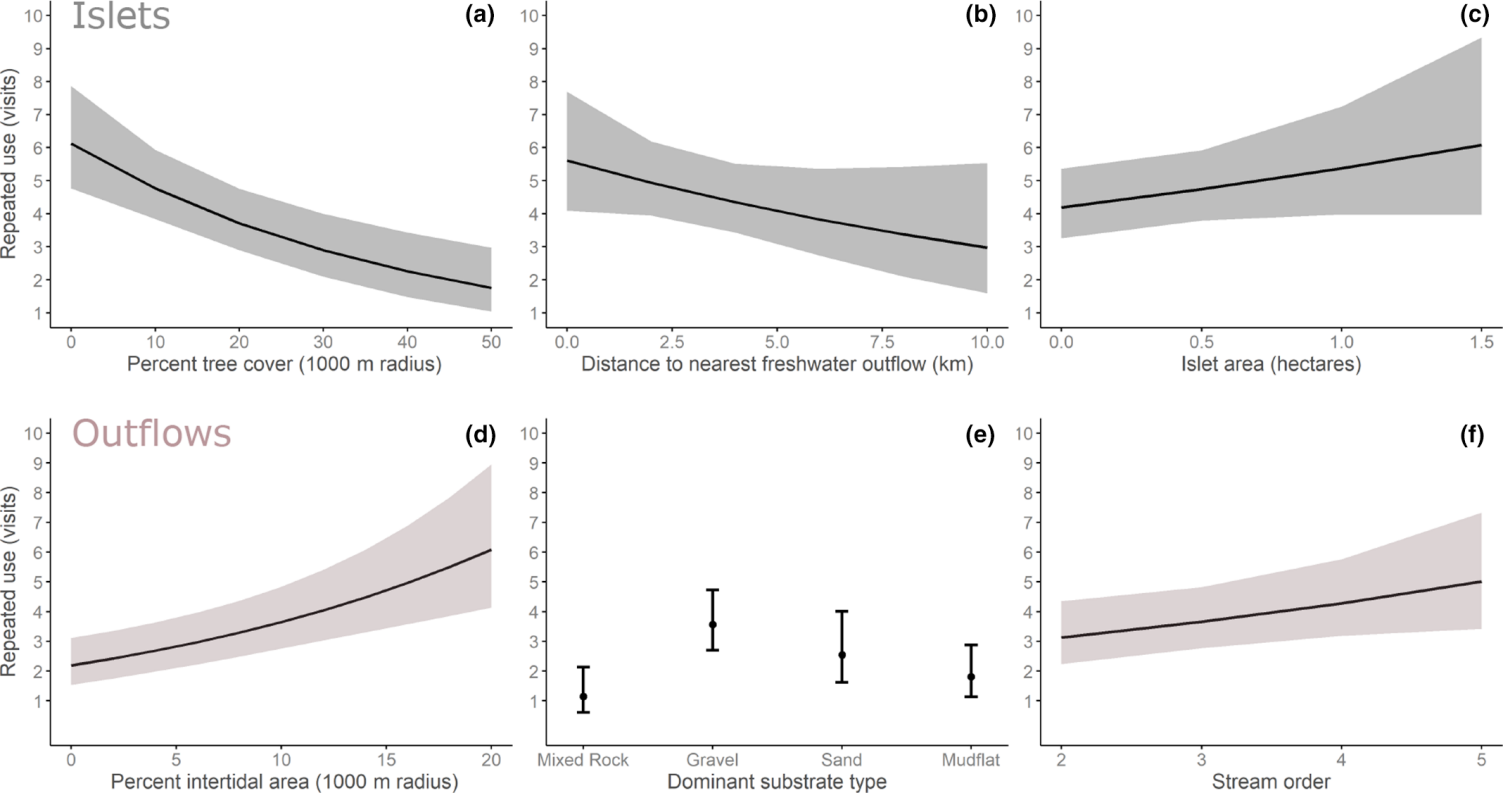
Model estimates and confidence intervals are from the top model in the candidate set for (a‐c) islets, and (d‐f) outflows. The islet model controls for the islet location relative to the center of an individual's core area (50% MCP).

After controlling for the location of islets relative to the center of an individual's core area, black oystercatchers returned more times to islets if there was less tree cover within a 1000 m radius, if the islet was larger, and if the islet was closer to a freshwater outflow. Five of the 16 models in the candidate set, examining variation in the use of islets, received substantial support (ΔAICc < 2.0; Table 2). The top‐ranked model included three terms: percent tree cover within a 1000 m radius (Tree_1000_), islet area (IsletArea), and distance to freshwater outflow (OutflowDist). This model received substantially more support than the null model and twice as much support as a more parsimonious model that only included the tree cover variable. Tree_1000_ was included in all well‐supported models (Ʃw_i_ = 1.0). The top model predictions estimate that the number of times an individual visited an islet decreased sixfold as the percent tree cover increased from 0% to 50% (*β* = −0.02, 95% CI = −0.04 to −0.01, Figure 5a).

**TABLE 2:** Candidate models evaluating the repeated use of islets by individual black oystercatchers (*n* = 19) over one year using satellite telemetry information.

Parameters	*K*	AICc	ΔAICc	Wt	Marginal *R* ^2^	Conditional *R* ^2^
**Islet features**						
Tree_1000_ + IsletArea + OutflowDist	9	1120.6	0.0	0.22	0.45	0.59
Tree_1000_ + Intertidal_1000_	8	1120.7	0.1	0.21	0.44	0.59
Tree_1000_ + IsletArea	8	1120.8	0.2	0.20	0.44	0.58
Tree_1000_	7	1121.9	1.2	0.12	0.44	0.58
Tree_1000_ + IsletArea + Intertidal_1000_ + OutflowDist	10	1122.6	2.0	0.08	0.45	0.59
Tree_1000_ + Intertidal_1000_ + OutflowDist	9	1122.8	2.2	0.07	0.45	0.58
Tree_1000_ + IsletArea + Intertidal_1000_	9	1122.9	2.3	0.07	0.44	0.59
Tree_1000_ + OutflowDist	8	1124.0	3.4	0.04	0.45	0.58
Null (CentroidDist + CentroidDist^2^)	6	1136.1	15.4	0.00	0.37	0.53

*Note*: The candidate set included 16 models but only those with <10.0 ΔAICc and the null model are shown. All models include an autocovariate term to correct for spatial dependence and the term “CentroidDist+CentroidDist^2^” to correct for an individual's nonlinear preference toward their core home range area. *K* is the number of parameters in the model, AICc is the Akaike's Information Criterion, ΔAICc is the differences between the model and the lowest AICc score, wt is the likelihood of each model in relation to all other models in the candidate set, marginal *R* ^2^ is the variance explained by fixed terms, and conditional *R* ^2^ is the variance explained by both the fixed and random factors.

The size of an islet term (IsletArea) was included in three of the five well‐supported models and received moderate support (Ʃw_i_ = 0.50), although the model‐averaged parameter estimate had confidence that bounded zero (*β* = 0.27, 95% CI = −0.05 to 0.60). The top model predictions estimated that the number of times an individual visited an islet increased by 50% as the islet area increased from 0.01 to 1.5 ha (Figure 5b). The distance to a freshwater outflow term (OutflowDist) was included in two of the five well‐supported models and received moderate support (Ʃw_i_ = 0.41), although the model‐averaged parameter estimate, again, had confidence that bounded zero (*β* = −0.07, 95% CI = −0.15 to 0.01). The top model estimated that the number of times an individual visited an islet decreased by 40% as the distance from the islet to a freshwater source increased from 1 to 10 km (Figure 5c). The remaining term, the amount of intertidal habitat within 1000 m radius of the islet (Intertidal_1000_), was included in one well‐supported model. However, this model received half of the support as the top model and the model‐averaged parameter estimate suggested that intertidal habitat had little impact on islet use by individuals (*β* = 0.0, 95% CI = −0.03 to 0.03).

#### 3.4.2 Outflows

Within their home range, individuals returned more to outflows if they were associated with larger streams, greater intertidal areas, and gravel substrates. Three out of 21 candidate models received strong support (Table 3). The top‐ranked model (w_i_ = 0.43) included three terms: intertidal area within a 1000 m radius (Intertidal_1000_), dominant substrate type (Substrate), and stream order (StreamOrder). This model received substantially more support than the null and twice as much support as the next best. The terms Intertidal_1000_ and Substrate were included in all well‐supported models (Ʃw_i_ = 1.0). The top model predictions estimated that the number of times an individual visited an outflow increased threefold as the intertidal area within a 1000 m radius increased from 10% to 20% (*β* = 0.05, 95% CI = 0.02 to 0.08, Figure 5d). Individuals returned most often to outflows with gravel‐dominated substrates, followed by sand, mudflat, and mixed rock (Figure 5e). Black oystercatchers did not use outflows with bedrock or man‐made substrate types. The top model estimated that the number of times an individual visited an outflow increased by 67% (Figure 5f) as the stream order increased from second to fifth order (*β* = 0.16, 95% CI = 0.01 to 0.31). The remaining terms, tree cover within a 1000 m radius (Tree_1000_) and Southness, received less support and model‐averaged parameter estimates for both terms bounded zero, suggesting that tree cover and aspect had little influence on the repeated use of outflows.

**TABLE 3:** Candidate models evaluating the repeated use of freshwater outflows on the shoreline by individual black oystercatchers (*n* = 16) over 1 year using satellite telemetry information.

Parameters	*K*	AICc	ΔAICc	Wt	Marginal *R* ^2^	Conditional *R* ^2^
**Outflow features**						
Intertidal_1000_ + Substrate + StreamOrder	8	255.4	0.0	0.43	0.49	0.61
Intertidal_1000_ + Substrate + Tree_1000_	8	256.4	1.0	0.26	0.49	0.61
Intertidal_1000_ + Substrate	7	256.7	1.3	0.23	0.45	0.58
Intertidal_1000_ + Substrate + Southness	8	259.1	3.7	0.07	0.45	0.57
Intertidal_1000_ + StreamOrder	5	265.3	9.8	0.00	0.24	0.37
Tree_1000_ + Substrate	7	265.4	10.0	0.00	0.38	0.41
Intertidal_1000_ + Tree_1000_	5	265.5	10.0	0.00	0.26	0.33
Null	3	275.9	20.5	0.00	0.00	NA

*Note*: The candidate set included 26 models but only those with <10.0 ΔAICc, and the null model are shown.

## 4 DISCUSSION

Habitat selection studies provide valuable insight into the requirements of individuals and populations, whether they are regarding the use of nest sites (Specht et al., 2020), overwintering sites (De La Cruz et al., 2014), migratory stopover sites (Belaire et al., 2014), foraging sites (Wilson, 1990), or roost sites (Peters & Otis, 2006). While habitat studies are often conducted at one study area or limited to a short period of time, technological advances have created new opportunities to study smaller animals for longer periods of time (Cagnacci et al., 2010). Over one annual cycle, we tracked the movements of 20 adult black oystercatchers at four coastal sites in British Columbia, Canada, using solar‐powered satellite devices. All tracked birds remained resident year‐round and used a relatively small proportion of the available shoreline. In general, black oystercatchers made extensive use of rocky islets and shoreline with fewer trees that are likely to provide a refuge from predators, and shoreline with large intertidal areas and freshwater outflows that would be expected to provide greater foraging rewards. However, preferences for these habitat characteristics were dependent on the season, time of day, and tidal state. Individuals made repeated use of specific islets and outflows within their home ranges, providing additional insight into the function of key habitat features.

Individuals are expected to exhibit preferences for habitat that reduces nest predation and offspring predation risk during the breeding season and habitat that enhances their own survival and future reproductive success during the nonbreeding season (Fontaine & Martin, 2006; Marra et al., 1998). Individual habitat selection decisions may also be less constrained during the nonbreeding season allowing studies to detect preferences for multiple habitat types. Consistent with these expectations, we found that habitat use during the breeding season was best predicted by a single characteristic linked to predation risk: tree cover within 250 m of the shoreline. Aligning with previous studies which have argued that black oystercatchers prefer to nest on rocky islets, outcrops, or gravel beaches with fewer surrounding trees (Dalgarno et al., 2017; McFarland, 2010; Weinstein et al., 2014). In contrast, we found that during the nonbreeding season black oystercatchers continued using safe areas but were more likely to use habitat that enhances foraging success. The greater‐than‐expected use of outflows was interesting because these are areas typically characterized by fine sediments deposited by streams, and black oystercatchers are considered a rocky intertidal specialist, preferring epifauna like mussels and limpets (Andres & Falxa, 2020; Hartwick, 1973; Tessler et al., 2014). The apparent seasonal difference in the use of outflows could be explained by (1) the spatial constraints related to reproductive duties of the breeding season, (2) greater foraging rewards during the nonbreeding season, (3) increased safety in the nonbreeding season when black oystercatchers gather in flocks (i.e., safety in numbers; Cresswell, 1994), or (4) higher thermoregulatory costs in winter influence selection for more sheltered but riskier sites (Yasué et al., 2003). Individuals made greater use of outflows associated with large stream orders, large intertidal areas, and gravel substrates that are likely to provide greater foraging rewards. Gravel substrates increase sediment stability and interstitial spaces (Cigarrıa & Fernandez, 2000) resulting in greater density/biomass of several clam species (Gillespie et al., 2001; Thompson, 1995). The introduced varnish clam (*Nuttallia obscurata*) readily consumed by black oystercatchers (Hollenberg & Demers, 2017) is found at high densities in the upper intertidal zone near freshwater sources (Dudas et al., 2007), which may explain why black oystercatchers were also more likely to visit outflows during higher tides. Our results provide evidence that outside the breeding season, freshwater outflows provide important habitat for black oystercatchers in B.C. and that clams may be more relevant to their diet than previously described.

Shorebirds move frequently between foraging sites and roost sites, reflecting the trade‐offs associated with each location and period of time (Lima & Dill, 1990). Theory suggests that dangerous foraging sites should only be used when safer foraging sites are unavailable, or the benefits of feeding outweigh the predation costs (Kotler et al., 1993). We therefore expected to detect preferences for black oystercatcher foraging habitat during low tides, when access to invertebrate prey is greatest. Large intertidal areas were associated with greater tree cover in our study areas (see Figure A2); therefore, these sites were likely perceived as more dangerous. Similarly, we expected to detect a preference for safe areas during high tides, when access to feeding is reduced, and at night, when visibility of predators is decreased. Black oystercatcher habitat use across diel and tidal cycles aligned with these predictions. The only time period we detected preferences for large intertidal areas was low daytime tides, indicating that foraging benefits outweighed the costs during this time. During high tides and the night, black oystercatchers used safer shoreline with less tree cover like rocky islets, which have been found to provide safe roost sites for a variety of shorebirds (Rogers et al., 2006; van der Kolk et al., 2020; Watts et al., 2021). Individuals also made greater use of islets that were closer to the center of their core home range, were larger, had less surrounding tree cover, and were closer to freshwater outflows. Taken together, our results highlight the importance of safe roost sites that provide both refuge from predators and proximity to foraging resources.

The year‐round home range size of resident animals is expected to vary depending on prey abundance (Kouba et al., 2017), distribution of foraging habitat (Legagneux et al., 2009), availability of safe nesting and roosting sites (Popa‐Lisseanu et al., 2009), and the state of the individual (sex, age, and reproductive status; Rolando, 2002). We found that home range size of black oystercatchers varied enormously by region, after controlling for breeding status and sex. Home range area was strongly correlated with the length of shoreline inside the home range, indicating that the geometry (or tortuosity) of the coastline did not explain the regional variation in space use. However, the length of shoreline used was not correlated with the length of shoreline within a home range (i.e., individuals all used a similar amount of shoreline) suggesting the distribution of suitable habitat explained the high variation in home range size (Rolando, 1998; Tarjan & Tinker, 2016). More specifically, black oystercatchers residing on the Sunshine Coast had much larger home ranges than individuals inhabiting Pacific Rim, Skidegate Inlet, and Masset Inlet. One plausible explanation for the relatively large home ranges of black oystercatchers on the Sunshine Coast is that anthropogenic disturbance alters the distribution and/ or use of suitable habitat in this region. Given the documented population declines of several coastal waterbirds in the Salish Sea (Ethier et al., 2020), further work examining anthropogenic effects on habitat use by marine birds is clearly warranted.

## 5 CONCLUSIONS

Black oystercatchers are considered an indicator of the health of rocky intertidal ecosystems (Tessler et al., 2014). Tracking the movements of individuals throughout the year illustrates that habitat use can be dynamic, even for specialist species restricted to one ecosystem type where individuals are sedentary and occupy permanent home ranges. Here, we show that habitat use by black oystercatchers reflects a trade‐off between predation risk and foraging rewards and consequently varies with season, the time of day, and tidal state. The extent to which habitat selection varied temporally highlights the importance of including the question of *when* as well as the question of *where* in habitat selection studies (McGarigal et al., 2016). By examining habitat use across multiple temporal scales, we were able to show that shoreline with freshwater outflows provides seasonally important foraging habitat for black oystercatchers. Individuals make repeated use of outflows that are likely to be the most productive and lead us to speculate that clams are a more important component of their diet than previously recognized. We also show that rocky islets, particularly those in close proximity to outflows, are a critical component of the habitat used by black oystercatchers highlighting the importance of refuges from predation that also allow access to nearby foraging resources in shaping their movement and habitat use throughout the year. While our coastlines face rapid anthropogenic and climate‐related change (Harley et al., 2006; Stocks & Vandeborne, 2017), our study suggests that the presence and habitat use of black oystercatchers is indicative of safe and productive habitat for other coastal wildlife.

## AUTHOR CONTRIBUTIONS

**Lena Ware:** Conceptualization (equal); data curation (equal); formal analysis (lead); methodology (lead); writing – original draft (lead); writing – review and editing (equal). **J. Mark Hipfner:** Conceptualization (equal); funding acquisition (equal); project administration (equal); resources (equal); writing – review and editing (equal). **David J. Green:** Conceptualization (equal); data curation (equal); funding acquisition (equal); resources (equal); supervision (equal); writing – original draft (equal); writing – review and editing (equal).

## FUNDING INFORMATION

Funding and support for this study was provided by Environment and Climate Change Canada, the Centre for Wildlife Ecology (CWE) at Simon Fraser University, an NSERC Graduate Scholarship to LW, and an NSERC Discovery Grant to DJG.

## CONFLICT OF INTEREST STATEMENT

The authors declare that they have no competing interests.

## Data Availability

Satellite tracking data used in the current study are available on Movebank (ID 696824680). More detail available in the corresponding thesis: https://summit.sfu.ca/item/34778.
